# The dataset for the stages of concerns of public-school teachers towards the use of e-learning platform: Malaysian context

**DOI:** 10.1016/j.dib.2020.105230

**Published:** 2020-02-04

**Authors:** Yong Zheng He, Farrah Dina Yusop

**Affiliations:** Department of Curriculum and Instructional Technology, Faculty of Education, University of Malaya, Malaysia

**Keywords:** Concerns based adoption model (CBAM), Reliability and validity, Covariance-based structural equation modelling (CBSEM), Confirmatory factor analysis (CFA)

## Abstract

This dataset contains demographic information of 355 respondents and a validated 32-items Stages of Concerns Questionnaire (SoCQ). The SoCQ questionnaire was developed based on the Concerns-Based Adoption Model (CBAM) which measures seven stages of concerns as the variables. They are *unconcerned, informational, personal, management, consequence, collaboration* and *refocusing*. The data was firstly tested with normality, followed by validity checking using confirmatory factor analysis (CFA). It is useful for policy makers and stakeholders to have a thorough understanding about teachers’ concerns on the use of the e-learning platform and thus, design suitable interventions to smoothen the adoption process of using the technology. This set of data could be used in a multi-racial developing country for more complex analyses.

**Table:** Specifications Table

Subject	Education
Specific subject area	Educational Technology
Type of data	Tables, Figures
How data were acquired	Through 32-items in the Stages of Concerns Questionnaire (SoCQ).
Data format	Raw, Analyzed
Parameters for data collection	The questionnaire includes these items: 1. Demographic information inclusive of gender, ethnicity, teaching experience, and frequency of using the e-learning platform per week (4 items) 2. Stages of concerns (32 items) 3. Open-ended question related to description of concerns in using the e-learning platform (1 item).
Description of data collection	The approval to collect data from public schools was obtained via the online Education Research Application System (eRAS 2.0). Upon approval, emails were sent out to the principals of 81 schools in the district of Petaling Perdana whose teachers have been pre-identified as active users of the e-learning platform. 355 teachers from 12 schools responded to the questionnaire, which gave a response rate of 80%. Data collection took about 2 weeks to complete.
Data source location	Institution: Primary and Secondary Public Schools City/Town/Region: Kuala Lumpur and Selangor Country: Malaysia Latitude and longitude (and GPS coordinates) for collected samples/data: Kuala Lumpur (3.1390° N, 101.6869° E), Selangor (3.0738° N, 101.5183° E)
Data accessibility	Repository name: Mendeley Data Data identification number: 10.17632/ztgbtpn36p.1 Direct URL to data: https://data.mendeley.com/datasets/ztgbtpn36p/1

**Table:** 

**Value of the Data** • The dataset provides an insight into the stages of concerns of public schools' teachers on the use of e-learning platform. • The availability of this open access dataset is essential for policy makers and stakeholders to have a thorough understanding about teachers' concerns on the use of the e-learning platform, so that suitable interventions could be introduced to smoothen the adoption process of the technology. • This dataset is also beneficial for other researchers in understanding the relationship between the demographic information of teachers and the Stages of Concerns on the use of e-learning platform.

## 1 Data description

This dataset contains variables’ definition (Table 1), different versions of the instrument throughout the validation process, a manual to interpret the stages of concerns [2] and a 32-items Stages of Concerns Questionnaire (SoCQ). The SoCQ was distributed to all the public-school teachers that responded to the email sent out by the researcher. The values of Skewness and Kurtosis were calculated for the normality test. Then, the convergent and discriminant validity of the instrument is established by Covariance-Based Structural Equation Modelling (CB-SEM). The data were accessible at https://data.mendeley.com/datasets/ztgbtpn36p/1. Fig. 3 shows the final fitted model.

**Table 1:** 7 Stages of Concerns and its definition.

	Stage	Definition
Unrelated	Unconcerned	User is not concerned or has little involvement with the technology.
Self	Informational	User knows about the technology but is unconcern about how the technology relates with his/her role. It might be another indication that the user is interested in understanding more about the technology.
	Personal	User knows about the technology and its requirement, and the user is aware about his/her effort to use the technology. The user begin to concern about his/her relationship with the technology.
Task	Management	User now focuses the on the process of using the innovation and how can the innovation affect his/her task.
Impact	Consequence	User is now concern about how the technology could impact his/her students.
	Collaboration	User begins to concern about working or using the innovation together other colleagues. The user is willing to learn more about the innovation.
	Refocusing	User is now focusing on exploring more possibilities about the technology.

**Fig. 1:**
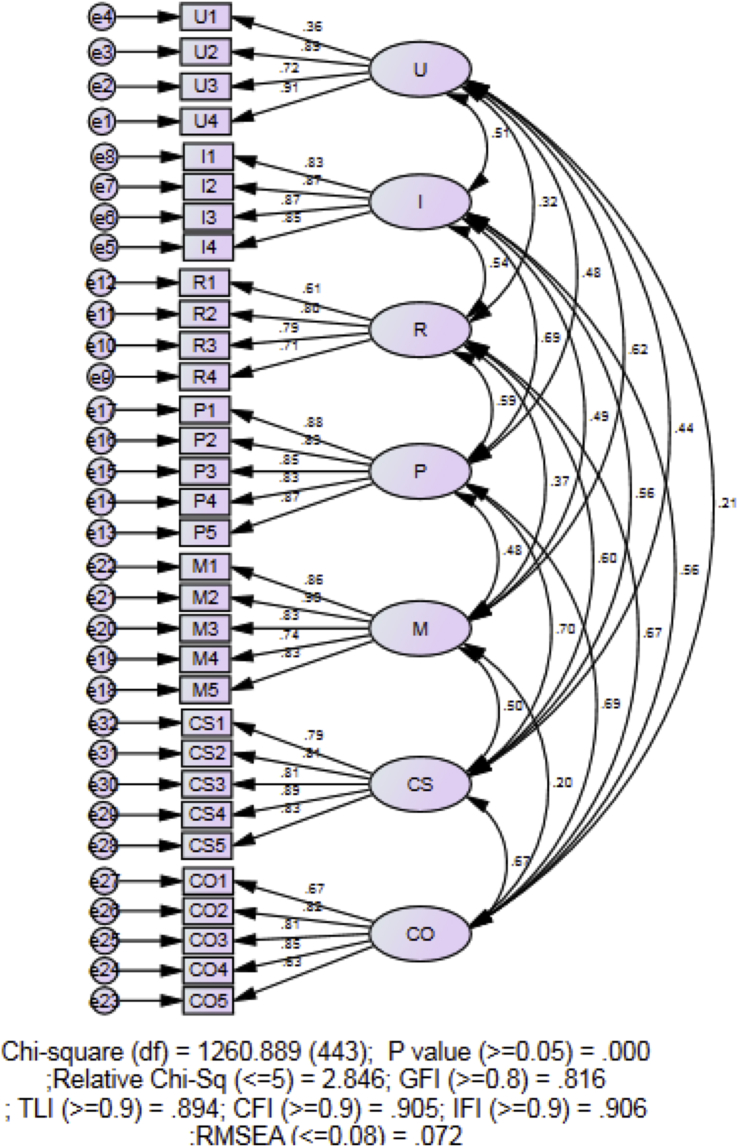
Initial order of measurement model.

**Fig. 2:**
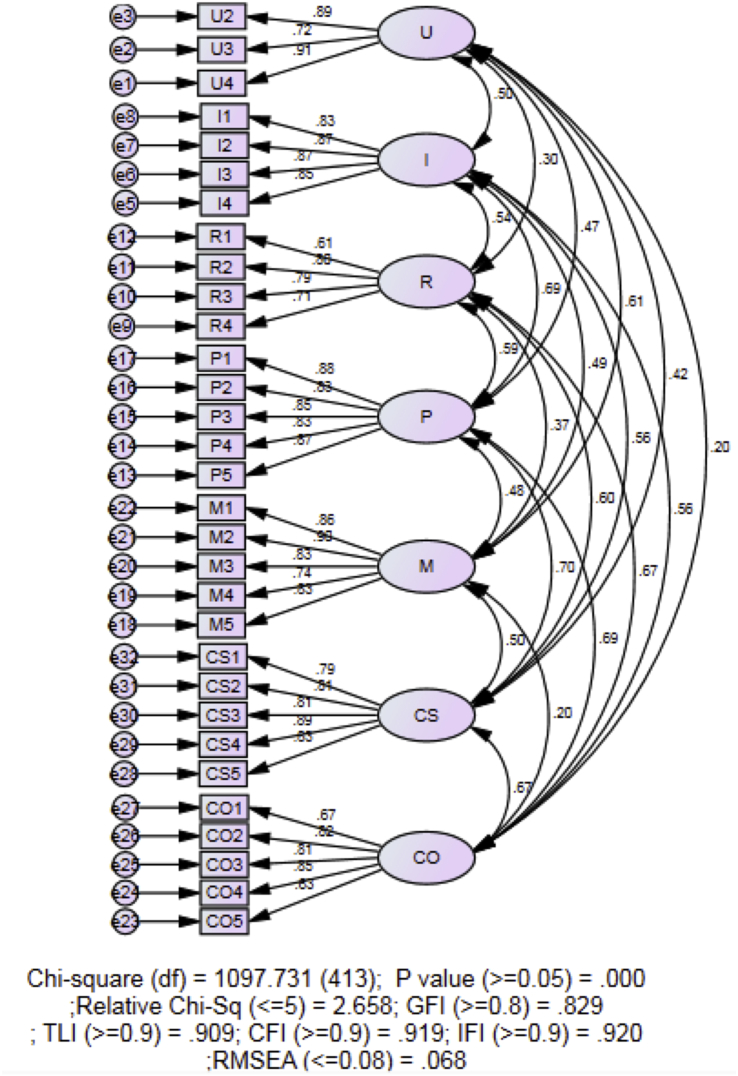
Measurement Model after removal of item U1.

**Fig. 3:**
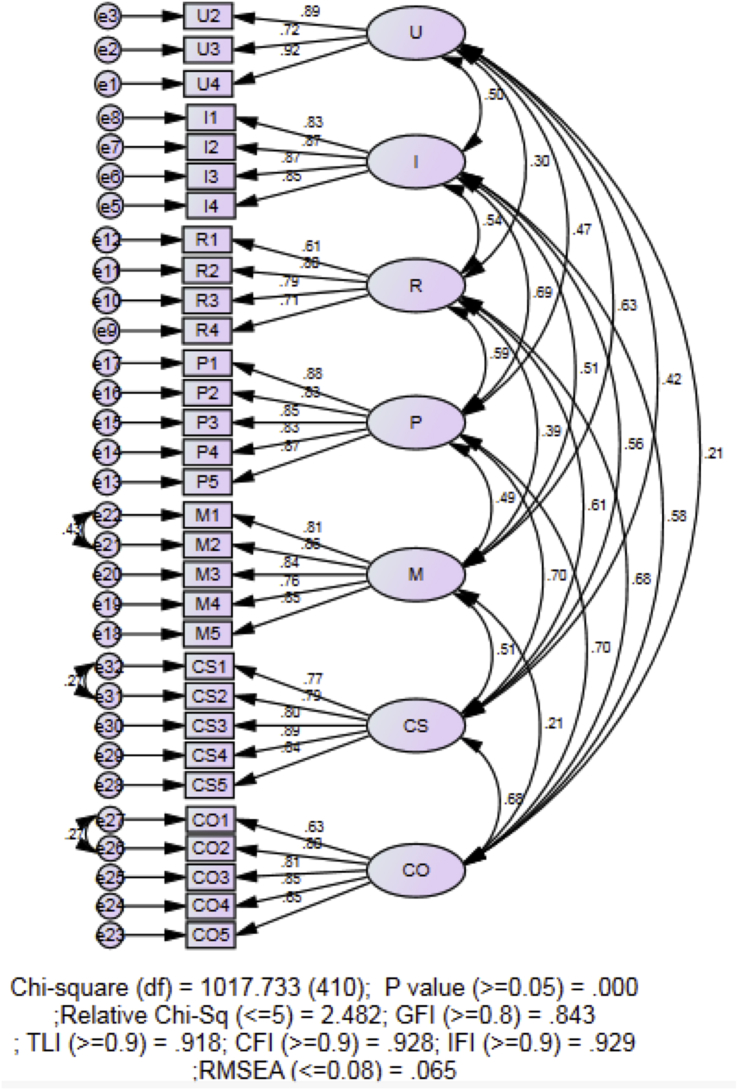
Final model.

## 2 Experimental design, materials, and methods

### 2.1 Concerned-based adoption model (CBAM)

There are three diagnostic dimensions in Concerns-Based Adoption Model. They are (i) stages of concerns, (ii) level of use, and (iii) innovation configurations. In this study, the SoCQ was adapted and distributed to the public schools’ teachers. The stages of concerns were initially conceptualized as three phases and user would move from one phase to another. The phases are: (i) unconcerned, (ii) Self-concerned, and (iii) concern with students [3]. The stages of concerns were then developed into different categories of concerns [4] and finally the revised stages of concerns (Table 1).

### 2.2 Normality test and confirmatory factor analysis (CFA)

After the data collection, normality test (Table 2) was conducted. Then the data is then tested for model fit. The initial order of measurement model analysis (Fig. 1) showed that $\chi^{2}$ (443, *N* = 355) = 1260.889, *p* < .000, $\chi^{2}/$DF = 2.846, GFI = 0.816; AGFI = 0.781, CFI = 0.905; IFI = 0.906, RMSEA = 0.072. The model is considered unfit because the value of TLI is less than the recommended 0.900.

**Table 2:** Values of Skewness and Kurtosis of all items.

Item	Skewness	Kurtosis	Item	Skewness	Kurtosis
U1	.007	-.611	M4	-.067	-.154
U2	-.379	-.626	M5	-.127	-.310
U3	-.010	-.606	CS1	-.160	-.191
U4	-.324	-.404	CS2	-.026	.021
I1	-.001	-.146	CS3	.006	.153
I2	-.212	-.199	CS4	-.036	-.072
I3	.009	-.208	CS5	-.090	.040
I4	-.185	.101	CO1	.441	-.147
P1	.002	-.450	CO2	.045	-.302
P2	-.088	-.183	CO3	-.050	-.028
P3	-.165	-.236	CO4	.038	.068
P4	-.262	.139	CO5	-.131	-.097
P5	-.327	.282	R1	-.192	-.286
M1	-.063	-.588	R2	.011	-.377
M2	-.128	-.210	R3	-.016	-.391
M3	.073	-.659	R4	.045	-.420

Item U1 was then removed due to low loading factor of 0.359 (Table 3) and also based on the modification indices recommended by AMOS (Fig. 2). Then, some of the error terms that belong to the same factor were covaried to see if the data fits the model. The final fitted model (Fig. 3) has all item loadings greater than 0.60 (Table 3), with $\chi^{2}$ (410, *N* = 355) = 1017.733, *p* < .000, $\chi^{2}/$DF = 2.482, GFI = 0.843; AGFI = 0.810, CFI = 0.928; IFI = 0.929, RMSEA = 0.065.

**Table 3:** Loadings of items.

Stages of Concerns	Items	Before Removal of Item U1	After Removal of Item U1	After Covaried Error terms
		(Estimate)	(Estimate)	(Estimate)
Unconcerned Stage	U1	0.359	Removed	Removed
	U2	0.887	0.888	0.886
	U3	0.723	0.721	0.72
	U4	0.91	0.915	0.917
Informational Stage	I1	0.834	0.834	0.834
	I2	0.866	0.867	0.866
	I3	0.872	0.872	0.871
	I4	0.85	0.85	0.85
Personal Stage	P1	0.879	0.879	0.878
	P2	0.831	0.831	0.83
	P3	0.848	0.848	0.848
	P4	0.828	0.828	0.828
	P5	0.872	0.872	0.873
Management Stage	M1	0.862	0.862	0.808
	M2	0.903	0.902	0.858
	M3	0.828	0.828	0.845
	M4	0.74	0.74	0.76
	M5	0.832	0.832	0.854
Consequence Stage	CS1	0.791	0.791	0.767
	CS2	0.811	0.811	0.79
	CS3	0.806	0.806	0.803
	CS4	0.886	0.886	0.893
	CS5	0.833	0.833	0.843
Collaboration Stage	CO1	0.669	0.669	0.633
	CO2	0.819	0.819	0.798
	CO3	0.806	0.806	0.806
	CO4	0.848	0.848	0.854
	CO5	0.634	0.634	0.646
Refocusing Stage	R1	0.607	0.607	0.606
	R2	0.805	0.805	0.804
	R3	0.792	0.792	0.792
	R4	0.707	0.707	0.709

These suggest that the data fits the model well based on the recommendations values (Table 4) of CMIN/df [5,6], GFI [7,8], CFI [6,9] and RMSEA [10].

**Table 4:** Recommended fit indices and the references.

Fit Indices	Authors/References	Recommended Criteria
CMIN/*df*	Marsh & Hocevar, 1985 Bentler, 1990	<5.0
GFI	Chau, 1997 Segars & Grover, 1993	>9.0
CFI	Bentler, 1990 Hatcher, 2013	>9.0
RMSEA	Byrne, 2001	<0.08

### 2.3 Reliability, convergent validity and discriminant validity

The values of composite reliability (CR), Average Variance Extracted (AVE), Maximum Shared Variance (MSV) and the loadings of the constructs (Table 5) were calculated using “Master Validity Tool” – an AMOS plugin.

**Table 5:** Values of CR, AVE and MSV using Master Validity Tool.

Stage of Concerns	CR	AVE	MSV	Convergent Validity		Discriminant Validity
				AVE > 0.5	CR > AVE	AVE > MSV
Unconcerned	0.882	0.715	0.395	Yes	Yes	Yes
Informational	0.916	0.732	0.482	Yes	Yes	Yes
Personal	0.930	0.725	0.493	Yes	Yes	Yes
Management	0.914	0.682	0.395	Yes	Yes	Yes
Consequence	0.911	0.673	0.493	Yes	Yes	Yes
Collaboration	0.866	0.567	0.486	Yes	Yes	Yes
Refocusing	0.820	0.536	0.466	Yes	Yes	Yes

The reliability of constructs with values between 0.82 and 0.93 are said to be satisfactory [11]. Since the values of AVE of all stages are greater than 0.5 and the AVE are all lesser than CR, convergent validity of the items is established [12,13]. The values of MSV are all found to be lesser than AVE (Table 5) and values at the square root of AVE (values at the diagonal) are higher than the correlation, showing the discriminant validity of the instrument (Table 6) [13,14].

**Table 6:** Values of Square root of AVE (values at the diagonal) and inter-construct correlation.

Stage	U	I	R	P	M	CO	CS
U	.846						
I	.500***	.856					
R	.302***	.545***	.732				
P	.472***	.694***	.594***	.852			
M	.628***	.512***	.389***	.495***	.826		
CO	.208***	.576***	.682***	.697***	.214***	.753	
CS	.417***	.564***	.608***	.702***	.510***	.685***	.82
